# Dihydromyricetin attenuates age-related macular degeneration: pharmacological effects and exploration of putative targets

**DOI:** 10.3389/fphar.2025.1588970

**Published:** 2025-08-21

**Authors:** Hongyu Zhou, Jianhui Pang, Baiyang Wu, Yanan Zhuang, Shenjun Li, Jing Jiang

**Affiliations:** ^1^ College of Pharmacy, Binzhou Medical University, Binzhou, Shandong, China; ^2^ Non-Clinical Research Department, RemeGen Co., Ltd, Yantai, Shandong, China

**Keywords:** age-related macular degeneration (AMD), dihydromyricetin (DHM), network analysis, molecular docking, putative targets

## Abstract

**Introduction:**

Age-related macular degeneration (AMD) is a leading cause of vision loss in older adults, with limited effective treatments available. This study aimed to investigate the pharmacological effects of dihydromyricetin (DHM) on AMD and to identify its putative pharmacological targets through network analysis and molecular docking approaches.

**Methods:**

*In vitro* experiments established an AMD model using sodium iodate (SI)-induced ARPE-19 cells, with CCK-8 assays determining 15 mM SI as the optimal modeling concentration and 100 μM DHM as the optimal treatment concentration. For *in vivo* validation, an AMD model was generated in C57 mice via tail vein injection of SI (30 mg/kg). Subsequent oral gavage with DHM (50 or 100 mg/kg) was administered. Integrated network analysis, molecular docking, and RT- qPCR validation were employed.

**Results:**

RT-qPCR analysis revealed that DHM reversed SI-induced aberrant expression of AMD-associated biomarkers (*ICAM-1, APOE, HTRA1, ABCA4*). Light microscopy and flow cytometry demonstrated DHM's significant mitigation of SI-triggered cellular morphological alterations and apoptosis (35% reduction). Western blot analysis further confirmed DHM-mediated suppression of apoptosis through regulation of p53, Bax, cleaved caspase-3, and Bcl-2 expression. High-dose DHM significantly attenuated retinal thinning (10.7% reduction), decreased pigment loss, and ameliorated structural disorganization in the outer nuclear layer (ONL). These analyses predicted seven putative targets implicated in functional categories including neurodegeneration, apoptosis, and DNA modification. Subsequent PPI network construction and GO/KEGG enrichment analyses revealed these targets' involvement in biological processes such as angiogenesis and extracellular matrix organization.

**Conclusion:**

In conclusion, the present study demonstrates that DHM can mitigate AMD-related damage in both *in vitro* and *in vivo* models, while predicting putative targets and signaling pathways through which DHM may exert its effects against AMD. These findings offer promising directions for the development of AMD therapies and lay the groundwork for further investigation into DHM as a candidate drug for treating and preventing AMD.

## 1 Introduction

Age-related macular degeneration (AMD) is a complex, multifactorial eye disease primarily affecting adults over 55 years old. It is a leading cause of vision loss in older adults, significantly impairing daily activities that require high-acuity central vision, such as reading and driving (Guymer and Campbell, 2023; Schmidt-Erfurth et al., 2014). With a global prevalence affecting approximately 196 million people worldwide, the prevalence of AMD is projected to rise to 288 million by 2040, a major public health concern (Fleckenstein et al., 2024). The disease is characterized by progressive degeneration of the retinal pigment epithelium (RPE), photoreceptor cells, and, in advanced stages, the choroidal vasculature (Blasiak, 2020).

AMD development is influenced by multiple risk factors, including age, genetic predisposition, and environmental triggers (Ambati and Fowler, 2012; Heesterbeek et al., 2020). Genetic studies have identified key pathogenic factors, such as variants in the *ARMS2*/*HTRA1* (Age-Related Maculopathy Susceptibility 2/HtrA Serine Peptidase 1) gene locus, which are strongly associated with an increased risk of neovascular AMD (Fritsche et al., 2016; DeWan et al., 1979). Additionally, complement-mediated activation and chronic inflammation have been linked to disease progression through *ICAM-1*(Intercellular Adhesion Molecule 1) secretion from choroidal endothelial cells, while drusen deposits beneath the RPE play a central role in initiating inflammatory responses (Skei et al., 2010). These extracellular deposits, composed of proteins, lipids, and cellular debris, can activate the complement system, recruiting immune cells that exacerbate RPE damage and retinal degeneration (Crabb et al., 2002; Wang et al., 2010; Mullins et al., 2000). Despite extensive research, the pathogenesis of AMD remains incompletely understood.

Oxidative stress and inflammation are recognized as critical factors driving disease progression (Datta et al., 2017; Heloterä and Kaarniranta, 2022). Oxidative stress, resulting from an imbalance between the production of reactive oxygen species (ROS) and cellular antioxidant defenses, is a key contributor to AMD. Persistent oxidative stress leads to mitochondrial dysfunction, lipid peroxidation, and protein/DNA damage, ultimately causing RPE apoptosis (Chan et al., 2019; Kaarniranta et al., 2019). The loss of RPE cells disrupts retinal structural integrity and function, accelerating the progression of AMD (Tang et al., 2021). Inflammation also plays a crucial role in AMD pathogenesis: drusen accumulation—extracellular deposits containing lipids, proteins, and cellular debris—beneath the RPE induces a chronic inflammatory response (Sakurada et al., 2023). This response involves complement system activation and immune cell recruitment, which further damage the RPE and surrounding structures (Crabb, 2014). Consequently, chronic inflammation and oxidative damage create a vicious cycle that promotes retinal degeneration and disease progression. Given the roles of oxidative stress and inflammation, targeting these pathways may slow the onset and progression of AMD.

Sodium iodate (SI) is a stable oxidizing agent widely used to induce retinal degeneration characterized by localized RPE loss and retinal toxicity in mice (NILSSON et al., 1977; Hariri et al., 2013), rats (Shen et al., 2010) and rabbits (Obata et al., 2005). Consequently, it serves as a well-established method for generating *in vitro* and *in vivo* AMD models.

Current AMD treatments primarily target the advanced neovascular form of the disease (Heloterä and Kaarniranta, 2022; Fabre et al., 2022). Anti-vascular endothelial growth factor (anti-VEGF) therapies inhibit pathological retinal angiogenesis, slowing vision loss progression (Girgis and Lee, 2023). However, these treatments require frequent intravitreal injections—an invasive approach associated with patient discomfort—and fail to address the underlying oxidative stress and inflammatory drivers of AMD. Critically, no effective therapies exist for dry AMD, which constitutes most cases and is defined by geographic atrophy of the RPE and photoreceptors (Stahl, 2020). These limitations underscore the urgent need for novel therapeutic strategies targeting upstream pathogenic mechanisms, particularly oxidative stress and inflammation.

Dihydromyricetin (DHM), a flavonoid metabolite derived from *Nekemias grossedentata* (Hand.-Mazz.) J. Wen and Z.L.Nie (Zeng et al., 2023), exhibits potent antioxidant, anti-inflammatory, and neuroprotective properties across multiple disease models. These include antitumour (Sun et al., 2019; Liu et al., 2014),antithrombotic (Jing and Li, 2020; Weng et al., 2017), and glucose/lipid regulatory effects (Liu et al., 2017; He et al., 2019), with particularly significant pharmacological effects in liver diseases (Chen et al., 2021; Chen et al., 2020). Studies demonstrate that DHM ameliorates dextran sulfate sodium-induced colitis in mice by modulating gut microbiota bile acid metabolism (Dong et al., 2021). Furthermore, DHM alleviates high glucose-induced oxidative stress and apoptosis in human retinal pigment epithelial cells via miR-34a downregulation, confirming its protective role against AMD *in vitro* (Li and Xiao, 2021). Collectively, these findings suggest DHM may mitigate AMD-associated oxidative stress and inflammation. By reducing Reactive Oxygen Species (ROS) production and regulating inflammatory pathways, DHM could preserve retinal structure and function, suggesting therapeutic potential for AMD management.

Network analysis combines systems biology principles to identify multi-target drug molecules by targeting key signaling nodes (Nogales et al., 2022; Zhao et al., 2023). When combined with molecular docking—which predicts optimal binding conformations between interacting molecules—this integrated approach effectively discovers putative targets for complex diseases (Li et al., 2022). Numerous studies have applied this strategy to uncover new drug targets (Zhou et al., 2022; Sun et al., 2023; Zhang et al., 2021), including isobavachin’s anti-hyperuricemic effect and fenugreek-derived multi-target therapies for diabetes (Luo J. et al., 2023; Luo W. et al., 2023).

This study aims to investigate the protective effects of DHM against sodium iodate-induced AMD both *in vitro* and *in vivo*, while predicting putative targets through network analysis and molecular docking techniques. This research approach investigated the pharmacological effects of DHM in AMD treatment and predicted putative targets using network analysis and molecular docking methodologies, thereby providing a foundation for developing novel therapeutic strategies targeting oxidative stress and inflammation in ophthalmic diseases with DHM.

## 2 Materials and methods

### 2.1 Cell culture and drugs treatment

The adult retinal pigment epithelial cell line ARPE-19 (ATCC) was routinely cultured in Dulbecco’s Modified Eagle Medium: Nutrient Mixture F-12 (DMEM/F12) (ATCC, Manassas, VA, United States, 30–2006) containing 10% fetal bovine serum (BI) and 100 U/mL penicillin/0.1 mg/mL streptomycin at 37°C in an incubator with 5% CO_2_. For model induction, SI was added to DMEM/F12 medium to a final concentration of 15 mM (Sigma-Aldrich, Saint Louis, MO, United States, S4007), and ARPE-19 cells were treated for 24 h to induce the AMD cell model. For drug treatment, DHM (0.1 mM) (MeilunBio, Dalian, LN, China, MB5834) and NaIO_3_ (15 mM) were added to DMEM/F12 medium and ARPE-19 cells were treated for 24 h.

### 2.2 Cell viability assay

The survival rate of ARPE-19 cells was determined using a Cell Counting Kit-8 (Beyotime, Shanghai, China, C0037). ARPE-19 cells were inoculated into 96-well culture plates (8 × 10^4^ cells/well) and treated with DHM or NaIO_3_ for 24 h, followed by incubation with CCK-8 reagent mixed 1:10 with fresh medium for 4 h, and absorbance was measured with an enzyme marker at 570 nm.

### 2.3 RNA extraction and RT-qPCR analysis

Total RNA was extracted from cultivated cells using Quick cell RNA extraction kit (SparkJade, Jinan, SD, China, AC0205-A). The cDNAs were synthesized using the PrimeScript™ RT reagent Kit with gDNA Eraser (Takara, Kyoto, Japan, 3735A). Quantitative RT-qPCR was performed with the StepOnePlus Real-Time PCR System (Thermo Fisher Scientific, Waltham, MA, United States). Ct values were calculated as: ∆Ct = Ct (target gene) – Ct (reference gene); ∆∆Ct = ∆Ct (treatment group) − ∆Ct (control group). The relative expression of the target genes was calculated using 2^−ΔΔCT^. All the primer sequences used in the experiment can be found in Supplementary Material 1.

### 2.4 Detection of cell death by flow cytometry

ARPE-19 cells were stained with Annexin V-FITC/PI using a commercial Annexin V-FITC/PI Apoptosis Detection Kit (Biolegend, Santiago, CA, United States, 640,914) according to the manufacturer’s instructions. ARPE-19 cells were treated with or without drugs for 24 h and then subjected to centrifugation to collect the cells, with 5 × 10^5^ cells per group. The cells were then incubated with Annexin V-FITC and PI, after which they were transferred to flow tubes and analyzed by flow cytometry (SONY, Kyoto, Japan, SA3800). Data analysis was performed using SA3800 software.

### 2.5 Immunoblotting

The cell pellet for protein expression detection was lysed by RIPA buffer (Beyotime, Shanghai, China, P0013B) containing a cocktail protease inhibitor (Beyotime, Shanghai, China, P1006). Protein concentration was quantified using the BCA method (Beyotime, Shanghai, China, P0009). According to the quantitative results, all the groups were unified at the same concentration. The lysate was then boiled at 95°C for 10 min. For SDS-PAGE, gradient gels (GenScript, Nanjing, JS, China, M00657) with a concentration range of 4%–20% were used to separate protein samples. The separated proteins were transferred onto a nitrocellulose membrane. The membrane was blocked with 5% milk in PBST at room temperature for 1 h. The corresponding primary antibody was used to incubate the blocked membrane overnight at 4 °C. After combining with secondary antibodies from the same species, protein bands were visualized by chemiluminescent substrates (Thermo Fisher Scientific, Waltham, MA, United States, 34,095). All the antibodies used in the experiment can be found in Supplementary Material 2.

### 2.6 *In vivo* animal model

This protocol of the animal experiment was approved by the Institutional Animal Care and Use Committee of Binzhou Medical University. Six-week-old male C57BL/6 J mice were purchased from Pengyue (Jinan, SD, China). The experimental mice were divided into four groups, each comprising eight mice, and their body weights were measured at designated time points throughout the dosing cycle. Mouse AMD model was prepared and each mouse received tail vein injection of sodium iodate (Sigma-Aldrich, Saint Louis, MA, United States, S4007) (30 mg/kg) (Wang et al., 2014). DHM was dissolved in 0.9% NaCl to prepare low-dose (50 mg/kg) and high-dose (100 mg/kg) solutions for post-modeling treatment (Dong et al., 2021; Xie et al., 2022; Yan et al., 2024). DHM was administered by gavage starting 3 days before SI injection, continuing for 8 days post-injection, with a total administration duration of 11 days.

### 2.7 Spectral domain optical coherence tomography (SD-OCT) and funduscopic examination

Mice were anesthetized by intraperitoneal injection of 1.25% tribromoethanol (AibeiBio, Nanjing, JS, China, M2910), followed by bilateral administration of 5 μL compound tropicamide ophthalmic solution (Novartis, Basel, Switzerland, S01FA51) to induce pupillary dilation; after achieving maximal mydriasis (>1.5 mm diameter), residual drops were removed and corneas protected with Carbomer 940 gel (Siccanove, Nice, France, CBM-940-OG) to prevent dehydration-induced opacity. Animals were immobilized in a stereotaxic platform with optimized ocular alignment for sequential imaging. Fundus photography was performed using a Micron III system (Phoenix Research Laboratories, Pleasanton, CA, United States), with pigment deposit area quantified using ImageJ (1.54i, NIH, Montgomery, MD, United States). High-resolution SD-OCT scans were acquired using radial B-scan patterns (Bioptigen, Morrisville, NC, United States). Retinal thickness (ILM-to-RPE distance) was quantified from optic disc-centered images via automated segmentation in InVivoVue Diver software (v3.1.7, Bioptigen).

### 2.8 Histological analysis of the retina

Enucleated mouse eyes were immediately fixed in 10% neutral buffered formalin (Sigma-Aldrich, Saint Louis, MO, United States, HT501640) at 4°C for 24 h to preserve retinal cytoarchitecture. Post-fixation, specimens were dehydrated through a graded ethanol series (Sinopharm, Shanghai, China, 64–17-5), cleared in xylene (Sinopharm, Shanghai, China, 1,330–20-7), and embedded in paraffin (Sinopharm, Shanghai, China, 8,002–74-2) per standard histological protocols. Serial sagittal sections containing optic nerve heads were cut at 3 μm thickness using a rotary microtome (Huasu Technology, Jinhua, ZJ, China, HS-3315), mounted on poly-L-lysine-coated slides (Epredia, Portsmouth, NH, United States, J2800AMNZ), and stained with hematoxylin and eosin (H&E) under controlled conditions: hematoxylin (Sigma-Aldrich, St. Louis, MO, United States, GHS316; 5 min), differentiation in 1% acid alcohol (30 s), eosin counterstain (Sigma-Aldrich, St. Louis, MO, United States, HT110316; 2 min). Bright-field images were acquired at × 200 magnification using an AxioScope 5 microscope (Carl Zeiss, Oberkochen, Germany, AxioScope5).

### 2.9 Target acquisition

DHM targets were retrieved from Swiss Target Prediction (http://www.swisstargetprediction.ch/) (Daina et al., 2019). AMD-related targets were collected from the OMIM database (http://omim.org/) (Hamosh et al., 2000) and GeneCards Database (http://www.genecards.org/). Then, the false-positive and duplicate targets were deleted and integrated. Finally, the targets of DHM and the targets of AMD were intersected to obtain the putative targets of DHM treating AMD.

### 2.10 Construction of the protein–protein interaction (PPI) network and functional enrichment analysis of genes in networks

Importing putative targets of DHM for AMD into GeneMANIA Database (https://genemania.org/) (Warde-Farley et al., 2010), and constructing protein-protein interaction networks. A cluster of genes highly associated with putative targets was obtained by constructing a network. Gene ontology (GO) and Kyoto Encyclopedia of Genes and Genomes (KEGG) gene sets were downloaded from the official GSEA website (https://www.gsea-msigdb.org/gsea/downloads.jsp) (Subramanian et al., 2005). Utilizing this as a foundation, all network genes were mapped to the background set and enriched through the R package clusterProfiler (version 3.14.3) to obtain gene set enrichment results.

### 2.11 Molecular docking of DHM with putative AMD targets

The 3D structure of DHM was obtained from the PubChem database (https://pubchem.ncbi.nlm.nih.gov/). The PDB files of the target protein structures were obtained from the RCSB PDB database (http://www.rcsb.org/) (Subramanian et al., 2005). Proteins were prepared using PyMOL 2.3.0 (https://www.pymol.org/). Docking was accomplished using AutoDock Vina 1.1.2 software (Trott and Olson, 2010). Subsequently, visual analysis was performed using PyMOL 2.3.0. The protein structures were prepared by removing water molecules and adding polar hydrogen atoms. The grid box was centered on the ligand-binding site with dimensions of 20 × 20 × 20 Å. A total of 10 conformational poses were generated for each ligand-target complex. The pose with the lowest binding energy (ΔG, kcal/mol) was selected for further analysis.

### 2.12 Statistical analyses

Statistical analyses and graphs were generated using GraphPad Prism 8.0.1 (San Diego, CA, United States). Normality was tested for all datasets. Data are presented as mean ± standard deviation (SD). For normally distributed data, differences among multiple groups were analyzed by one-way ANOVA; for non-normally distributed data, the Kruskal–Wallis test was used, supplemented by homogeneity of variance testing. All data met homogeneity of variance requirements. Thus, pairwise comparisons for normally distributed data used Tukey’s test, while Dunn’s test was applied to non-normally distributed data. Statistical significance between groups was determined by these post-hoc tests (*p < 0.05, **p < 0.01, ***p < 0.001).

## 3 Results

### 3.1 *In Vitro* verification of DHM’s inhibition of AMD progression

SI-induced RPE cell injury is a well-established *in vitro* AMD model. Reported SI concentrations vary across studies due to methodological differences.

**FIGURE 1:**
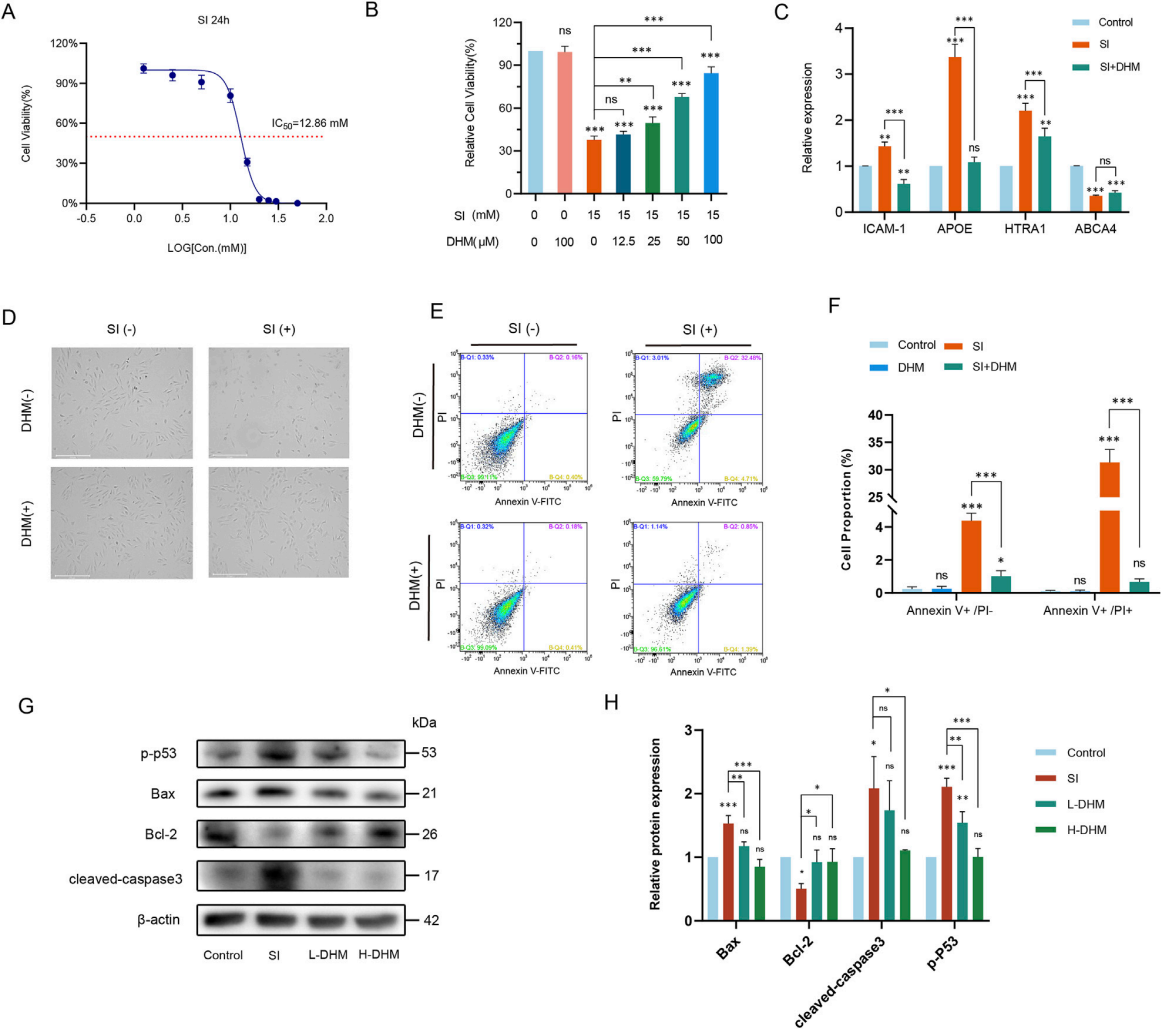
DHM attenuates sodium iodate-induced AMD pathology *in vitro*. **(A)** IC_50_ determination of SI in ARPE-19 cells via CCK-8 assay. **(B)** Cell viability after 24 h co-treatment with 15 mM SI and DHM (CCK-8 assay). **(C)** mRNA expression of AMD-associated markers (*ICAM-1*, *APOE*, *HTRA1*, *ABCA4*) by RT-qPCR. **(D)** Morphological changes in ARPE-19 cells post 24 h treatment. **(E)** Apoptosis analysis by Annexin V-FITC/PI flow cytometry. **(F)** Quantification of flow cytometry data. **(G)** Western blot analysis of apoptosis-related proteins (p-p53, Bax, cleaved caspase-3, Bcl-2). **(H)** Densitometric quantification of protein expression. Data were analyzed using one-way analysis of variance (ANOVA) followed by Tukey’s post-hoc test and are presented as mean ± standard deviation (*n* = 3 per group). ns: *p* > 0.05, *p < 0.05, **p < 0.01, ***p < 0.001. Significance symbols: Above bars: vs. untreated control; Between bars: vs. SI model group.

First, we determined SI cytotoxicity in ARPE-19 cells under our experimental conditions. The half-maximal inhibitory concentration (IC_50_) of SI was 12.86 mM (CCK-8 assay; Figure 1A). For model consistency, 15 mM SI was selected for subsequent experiments. Six DHM concentrations (0, 12.5, 25, 50, 100, 200 μM) were tested based on prior studies (Dong et al., 2021; Li and Xiao, 2021). DHM’s protective effect against SI-induced injury was assessed via CCK-8 assay across six concentrations. Under the treatment of 100 μM DHM, the cell viability was about 86%, and with a 120% improvement compared to the SI-only group. Thus, 100 μM DHM was selected as the optimal concentration (Figure 1B).

The mRNA expression of four AMD-associated markers—*ICAM-1*, *APOE*, *HTRA1*, and *ABCA4*—was analyzed by RT-qPCR (Yang et al., 2016; Vessey et al., 2022; Pan et al., 2023; Farnoodian et al., 2022). Compared to untreated controls, modeling with 15 mM SI significantly upregulated *ICAM-1*, *APOE*, and *HTRA1* transcripts while downregulating *ABCA4*. Notably, 100 μM DHM treatment reversed these alterations, restoring marker expression toward baseline levels relative to the model group (Figure 1C).

Light microscopy revealed comparable cellular morphology between the DHM-only (100 μM) and untreated control groups. Following 15 mM SI treatment, ARPE-19 cells exhibited pronounced morphological alterations and extensive cell death. However, DHM co-treatment markedly reduced SI-induced cell death and restored normal morphology (Figure 1D). Flow cytometric analysis demonstrated that 100 μM DHM alone induced no significant cytotoxicity relative to negative controls. After sodium iodate treatment, approximately 5% of the cells were FITC Annexin V positive and PI negative (early apoptosis), and approximately 30% of the cells were FITC Annexin V and PI positive (terminal apoptosis or necrosis). DHM co-treatment significantly reduced both apoptotic populations and normalized cellular viability (Figures 1E,F).

To elucidate DHM’s role in SI-induced apoptosis, Western blot analysis revealed that SI upregulated phosphorylated p53 (p-p53), Bax, and cleaved caspase-3 while downregulating the anti-apoptotic protein Bcl-2 relative to controls. Critically, both high and low DHM concentrations attenuated these alterations: suppressing p-p53, Bax, and cleaved caspase-3 overexpression, and restoring Bcl-2 levels. Thus, DHM inhibited SI-triggered apoptosis in ARPE-19 cells (Figures 1G,H).

### 3.2 *In Vivo* pharmacological effects of DHM in AMD mouse models

**FIGURE 2:**
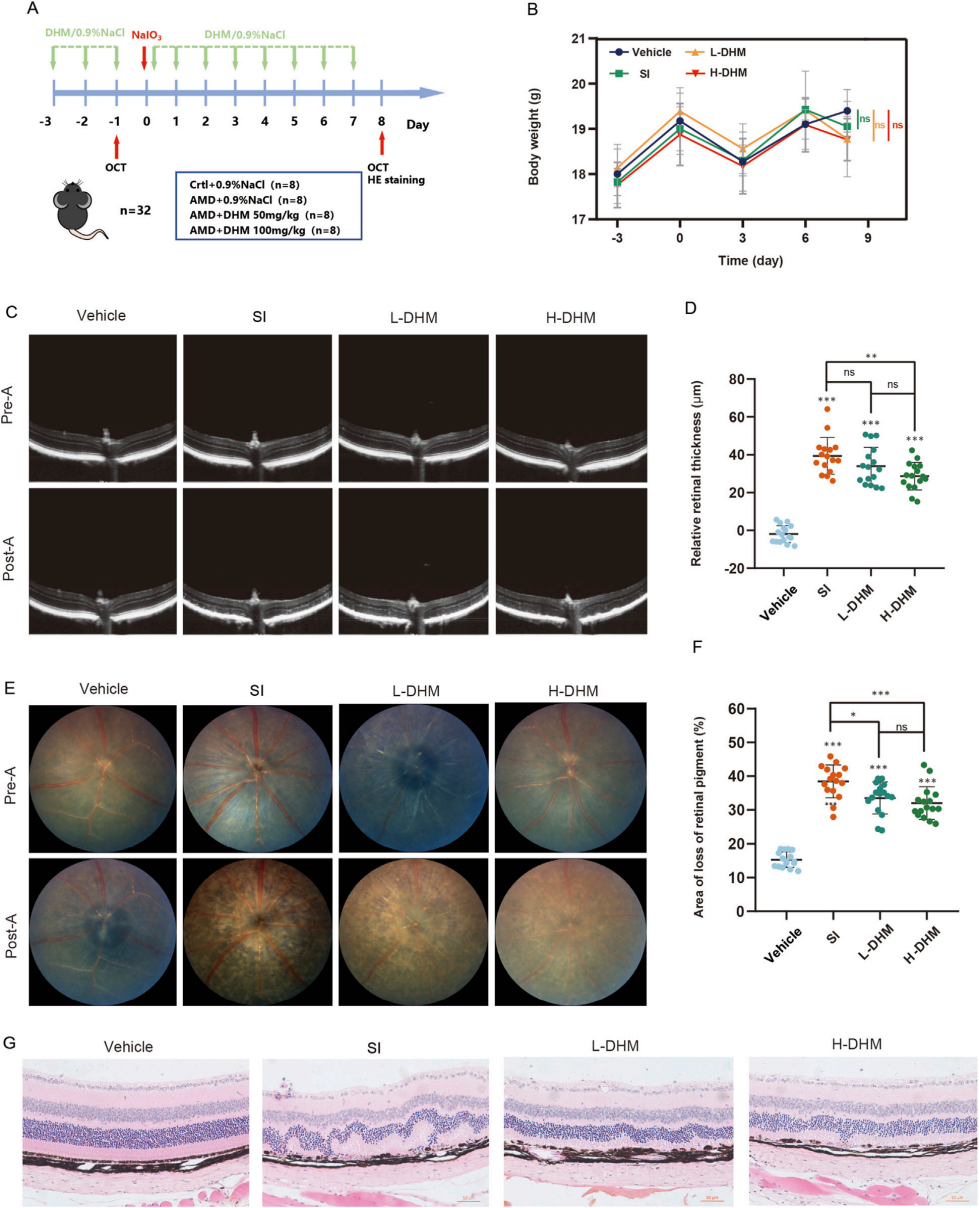
DHM alleviates AMD pathology *in vivo*. **(A)** Experimental timeline of DHM and SI administration in C57 mice. **(B)** Body weight changes across treatment groups during the study. **(C)** Retinal thickness measurements by SD-OCT pre- and post-treatment. **(D)** Quantitative analysis of retinal thickness changes. **(E)** Representative fundus images showing retinal pigment status. **(F)** Quantification of retinal pigment loss improvement. **(G)** H&E staining of retinal sections (The scale bar = 50 μm, and the magnification is ×200). Data were analyzed using one-way analysis of variance (ANOVA) followed by Tukey’s post-hoc test and are presented as mean ± SD (*n* = 16 per group). ns: *p* > 0.05, *p < 0.05, **p < 0.01, ***p < 0.001. Significance symbols: Above bars: vs. untreated control; Between bars: vs. SI model group.

To validate DHM responses in murine AMD, we established an AMD model in C57 mice by injecting SI (30 mg/kg) into the tail vein. Mice received oral DHM (50 or 100 mg/kg) for 3 days before SI injection, and treatment was continued for 7 days post-injection (Figure 2A). Body weights were measured every 3 days throughout the experiment. No significant differences in body weight were observed among the four groups, and no adverse effects (e.g., piloerection, convulsions, motor disorders, or abnormal somnolence) occurred (Figure 2B). Retinal thickness (from the ILM to the ROST) was measured by fundus photography and SD-OCT before DHM administration and at the end of the treatment cycle. The SI model group showed a significant reduction in retinal thickness (approximately 39.4 μm). Both DHM doses attenuated this reduction, but only the high-dose group (100 mg/kg) showed statistically significant effects, with an average improvement of 10.7 μm (27% improvement rate) (Figures 2C,D). Fundus examination revealed that DHM reduced retinal pigment loss by approximately 5% (low-dose) and 6.5% (high-dose) (Figures 2E,F). Furthermore, HE staining showed pronounced disorganization in the outer nuclear layer (ONL) of SI-treated mice versus controls, while DHM treatment reduced ONL disorganization (Figure 2G). These findings indicate that oral DHM markedly attenuates SI-induced retinal injury.

### 3.3 Prediction of putative DHM targets against AMD

**FIGURE 3:**
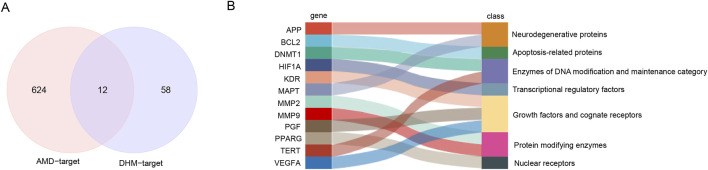
Predicting putative targets of DHM for alleviating AMD. **(A)** Venn diagram of candidate targets identified through multi-database screening. **(B)** Functional enrichment analysis of the 12 putative targets.

AMD-related targets were collected from OMIM (gene map) and GeneCards (relevance score >10) and a total of 636 AMD-related target genes were obtained after deletion of duplicates. The DHM drug target genes were collated from the Swiss Target Prediction (Probability*>0). Resulting in a total of 70. AMD disease target genes and DHM drug target genes were taken and intersection was taken to obtain putative targets of DHM against AMD. Eventually, a target gene set of 12 targets was obtained (Figure 3A). These targets can be broadly classified into seven categories according to their functions, including neurodegenerative proteins (*APP*, *MAPT*), apoptosis-related proteins (*BCL2*), Enzymes of DNA Modification and Maintenance Category (*DNMT1, TERT*), Transcriptional regulatory factors (*HIF1A*), Growth factors and cognate receptors (*KDR*, *PGF*, *VEGFA*), protein modifying enzymes (*MMP2*, *MMP9*) and nuclear receptors (*PPARG*) (Figure 3B).

### 3.4 PPI network construction and Functional Enrichment analysis of target gene sets

**FIGURE 4:**
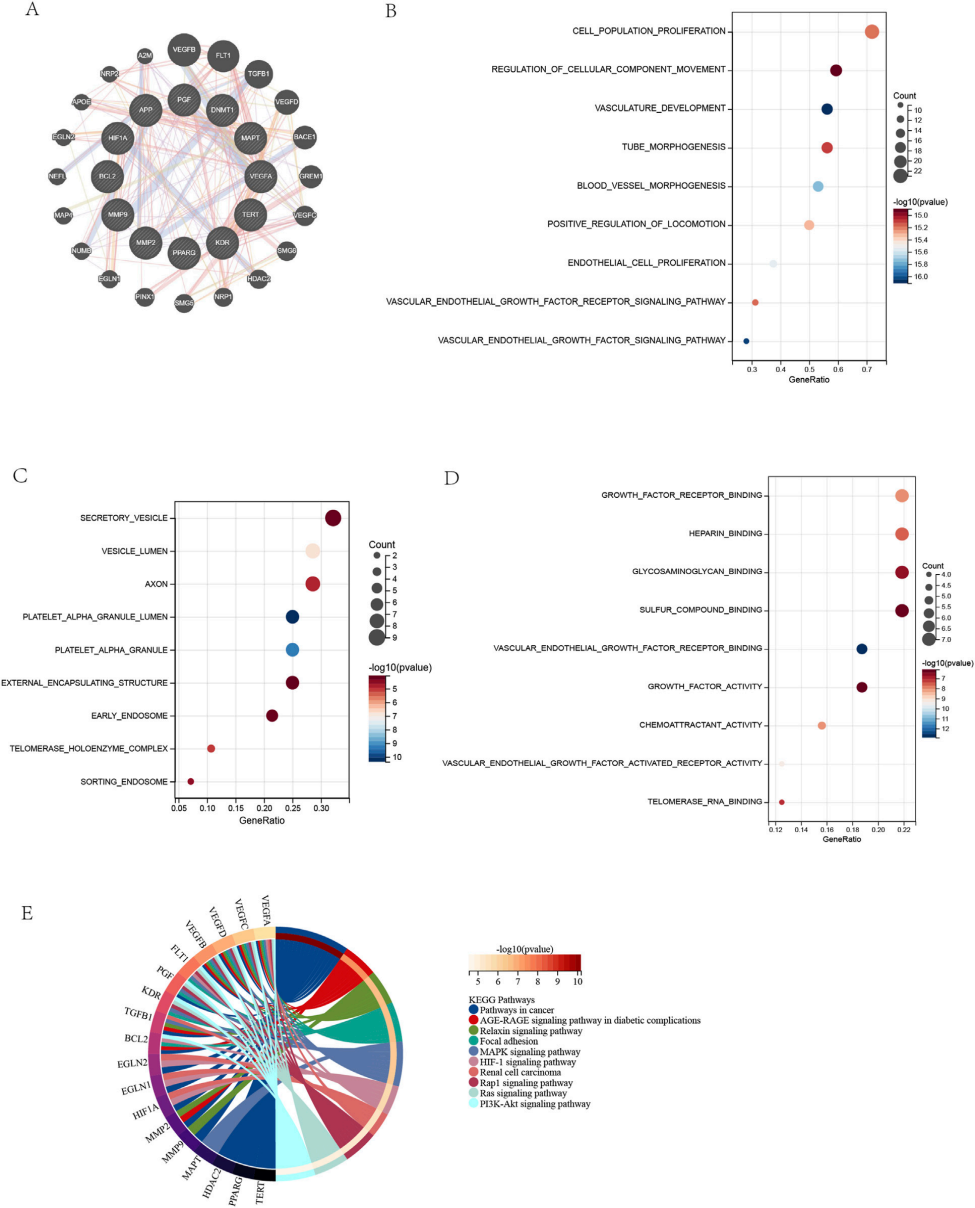
Protein-Protein Interaction Network and Functional Enrichment of Target Genes. **(A)** PPI network of DHM-AMD targets (12 core +20 related genes). **(B)** GO: Biological Process enrichment (top 10 terms). **(C)** GO: Cellular Component enrichment (top 10 terms). **(D)** GO: Molecular Function enrichment (top 10 terms). **(E)** KEGG pathway enrichment (top 10 terms). Enrichment criteria: FDR <0.05 (Benjamini and Hochberg correction).

A protein-protein interaction (PPI) network comprising 12 core targets and 20 functionally related genes was constructed using GeneMANIA, visually presenting interactions among target genes (Figure 4A). The full set of 32 genes underwent GO and KEGG enrichment analyses. GO biological process analysis revealed top 10 pathways enriched in angiogenesis regulation, including vascular endothelial growth factor signaling, vasculature development, and endothelial cell proliferation, indicating core targets’ roles in vascular morphogenesis and proliferation control (Yan et al., 2024; Gui et al., 2023) (Figure 4B). GO cellular component analysis showed enrichment in subcellular structures: platelet α granule lumen, telomerase holoenzyme complex, collagen-containing extracellular matrix, secretory vesicles, and axons, suggesting involvement in vesicle transport, extracellular matrix organization, and neuronal maintenance (Figure 4C). Notably, VEGF from platelet α granules may promote angiogenesis via vesicular transport (Manne et al., 2017). GO molecular function terms included VEGF receptor binding, growth factor activity, and chemotactic factor activity, implying regulation of angiogenesis and immune cell recruitment (Figure 4D) (Sharma et al., 2012; Rosenfeld, 2011). DHM may inhibit angiogenesis by blocking VEGF receptor binding or chemokine activity, aligning with anti-angiogenic therapies (e.g., bevacizumab). KEGG pathway enrichment highlighted Pathways in Cancer, MAPK signaling, and PI3K-Akt signaling, linking targets to oncogenic signaling and metabolic disease mechanisms (Figure 4E) (Wu et al., 2025; Muraleva and Kolosova, 2023). Complete GO/KEGG results are in Supplementary Material 3.

### 3.5 *In Vitro* assessment of DHM effects on target genes

**FIGURE 5:**
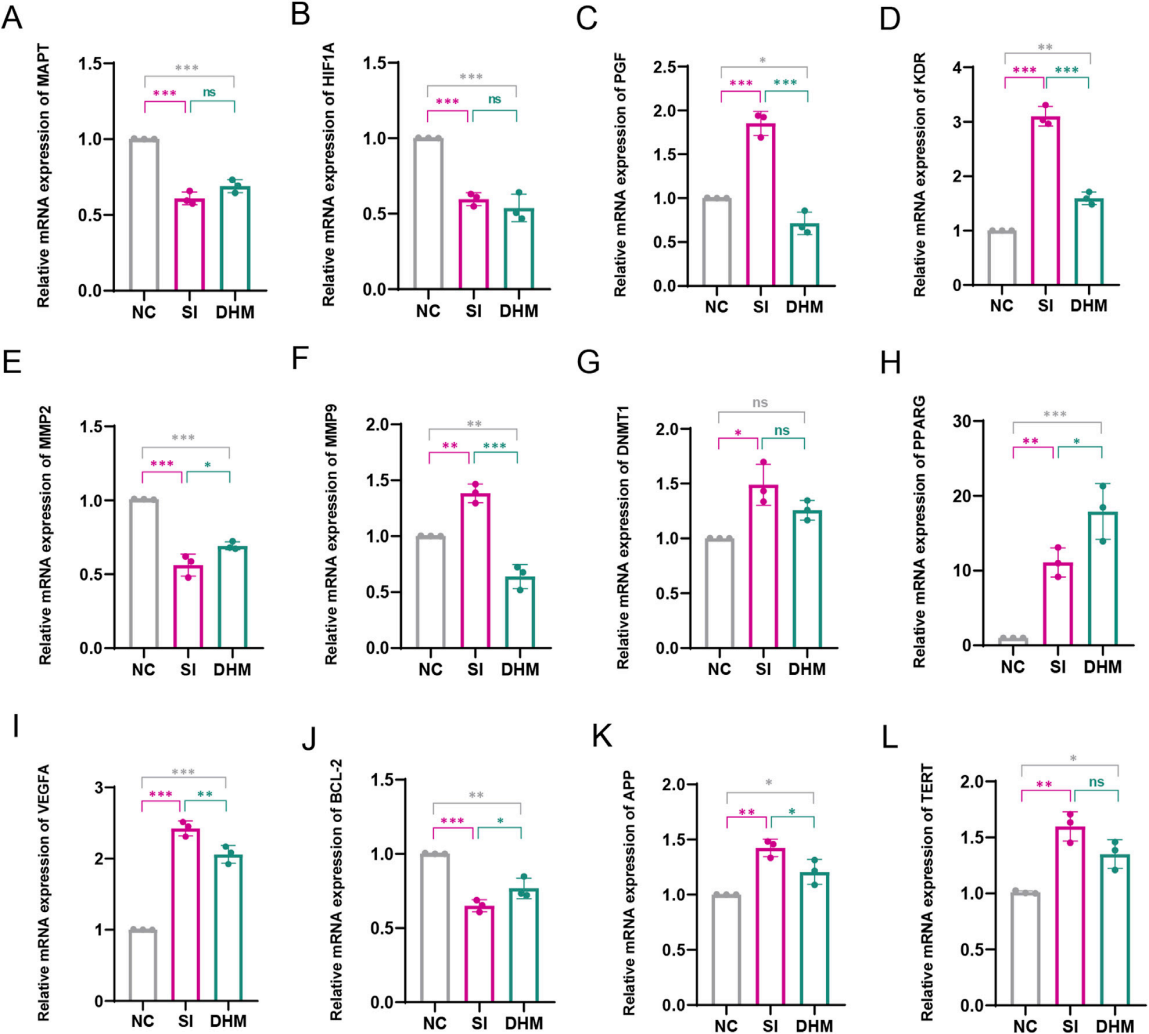
Effect of DHM on target proteins. The mRNA expression levels of *MAPT* **(A)**, *HIF1A* **(B)**, *PGF* **(C)**, *KDR* **(D)**, *MMP2* **(E)**, *MMP9* **(F)**, *DNMT1* **(G)**, *PPARG* **(H)**, *VEGFA* **(I)**, *BCL2* **(J)**, *APP* **(K)**, *TERT* **(L)**. Data in Figure G (non-normally distributed) were analyzed by Kruskal–Wallis with Dunn’s test; other data (normally distributed) were assessed by one-way ANOVA with Tukey’s test. Values represent mean ± SD (*n* = 3 per group). ns: *p* > 0.05, *p < 0.05, **p < 0.01, ***p < 0.001. Significance symbols: Above bars: vs. untreated control; Between bars: vs. SI model group.

RT-qPCR was used to detect the mRNA expression levels of target proteins in NC, SI and DHM treatment groups. *PGF*, *KDR*, *MMP9*, *VEGFA* and *APP* were significantly upregulated in the SI group, and significantly downregulated after DHM treatment. *MMP2*, *BCL2* were significantly downregulated in the SI group, and significantly upregulated after DHM treatment. *MAPT*, *HIF1A*, *DNMT1*, *PPARG* and *TERT* showed no obvious alleviating effects after DHM treatment compared with the SI treatment group. (Figures 5A–L). Notably, some predicted targets have been experimentally validated as pivotal regulators in AMD pathological processes. For example, *MMP2* supplementation has been shown to ameliorate drusen accumulation, while *MMP9* and *APP* have been identified as potential AMD biomarkers (Lynn et al., 2022; Hussain et al., 2011). Co-inhibition of *VEGFA* and *PGF* has been demonstrated to restrict retinal neovascularization in mice (Balser et al., 2019).

### 3.6 Molecular docking analysis of DHM-Target binding

**FIGURE 6:**
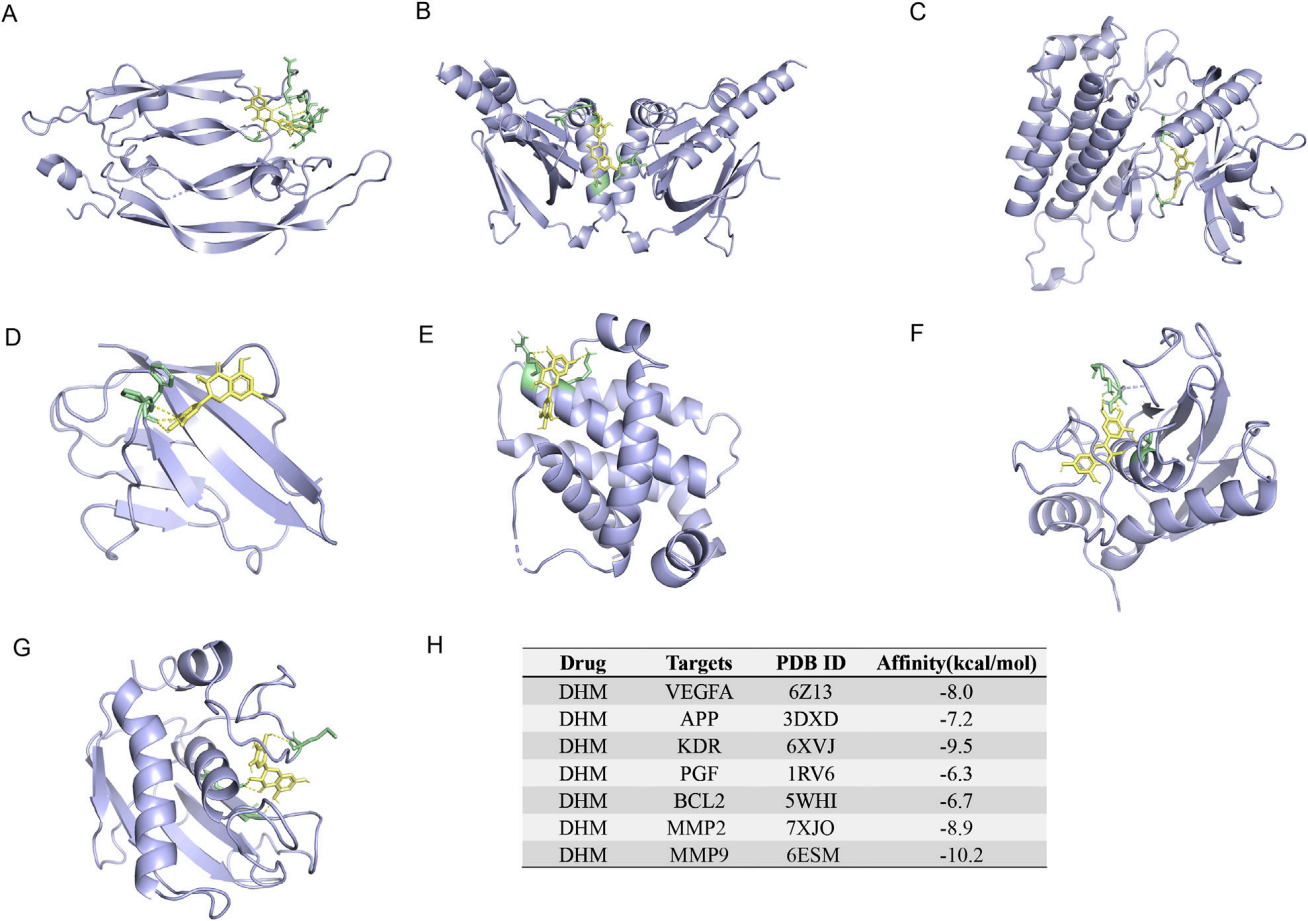
The 3-dimensional map of the binding sites between DHM and target proteins. **(A)** *VEGFA*, **(B)** *APP*, **(C)** *KDR*, **(D)** *PGF*, **(E)** *BCL2*, **(F)** *MMP2*, **(G)** *MMP9*. **(H)** Docking information and binding capacity of individual proteins.

RT-qPCR results confirmed DHM’s inhibition of aberrant mRNA expression in these seven targets. Therefore, molecular docking between DHM and the targets was performed using AutoDockTools-1.5.7 to validate binding affinity. The lowest-energy docking poses were retained for analysis. DHM exhibited the strongest binding to *MMP9* (binding energy = −10.2 kcal/mol), followed by *KDR*, *MMP2*, *VEGFA*, *APP*, *BCL2*, and *PGF* (Figure 6H). Molecular interactions at binding sites are visualized in Figures 6A–G.

## 4 Discussion

This study demonstrates that DHM effectively mitigates AMD pathology, as evidenced by protective effects in both *in vitro* and *in vivo* models. *In vitro*, DHM significantly suppressed SI-induced apoptosis in ARPE-19 cells, preserving cellular integrity against oxidative damage. *In vivo*, DHM alleviated key retinal degeneration markers in SI-induced AMD mice: attenuating retinal thinning, reducing pigment loss, and restoring ONL organization. These dose-dependent pharmacological effects (maximal at high doses) suggest DHM’s potential for AMD prevention and treatment.

The study builds on previous research, such as the work by Li and Xiao (2021), which demonstrated DHM’s protective effects on high glucose-induced oxidative stress and apoptosis in RPE cells. However, Li et al.‘s study was limited to *in vitro* experiments, leaving DHM’s *in vivo* effects unexamined. Beyond confirming the protective effect of DHM against RPE cell apoptosis *in vitro*—mediated through inhibition of the p53-dependent Bcl-2 apoptotic pathway—this study extended these findings to an *in vivo* setting. We established a sodium iodate-induced AMD murine model, enabling comprehensive evaluation of DHM’s therapeutic potential. This study supports DHM as a prospective AMD pharmacological agent by demonstrating its attenuation of AMD progression in a human-mirroring animal model.

Current AMD therapies (e.g., anti-VEGF injections) primarily slow neovascular AMD progression but face limitations (Rosenfeld, 2011; Markham, 2019). These therapies require frequent intravitreal injections, which can be invasive, uncomfortable, and costly for patients. Furthermore, they do not address the underlying oxidative and inflammatory processes that contribute to both neovascular and dry forms of AMD. As a result, many patients with the dry form of the disease, which accounts for a majority of cases, have no effective treatment options available. DHM, as demonstrated here, offers a promising alternative. By simultaneously modulating oxidative stress and inflammation, DHM addresses core AMD pathogenesis. Its oral bioavailability further enhances clinical potential, potentially reducing treatment burden and costs while providing systemic protection. However, despite the convenience of oral administration, DHM’s bioavailability and long-term toxicity require validation in large animal models. In the future, phase I clinical trials should be conducted to evaluate its safety.

Network analysis predicted 12 putative targets associated with DHM’s counteraction of AMD, all of which represent key pathogenic pathways involved in angiogenesis, apoptosis regulation, and oxidative stress response. Seven targets were preliminarily validated through quantification of mRNA expression *in vitro*, showing significant modulation upon DHM treatment. Molecular docking analysis demonstrated robust binding affinities between DHM and these targets—including *MMP9*, *VEGFA*, and *BCL2*—suggesting that DHM may exert its pharmacological effects by modulating relevant signaling pathways through interactions with these critical proteins.

Notably, this study has several limitations that should be acknowledged. First, as a flavonoid metabolite, DHM may act as a pan-assay interfering substance (PAINS), exerting non-specific effects via mechanisms such as redox cycling or metal chelation (Bolz et al., 2021; Lagorce et al., 2017). While our *in vitro*, *in vivo*, and target-binding data support specific pharmacological effects, further studies (e.g., with structural analogs of DHM lacking PAINS-associated moieties) are needed to confirm target specificity. Second, the sodium iodate tail vein injection mouse model represents a validated and widely used approach for establishing experimental AMD, specifically mimicking the geographic atrophy stage of advanced dry AMD (Wang et al., 2014; Kiuchi et al., 2002). Its primary advantage lies in the precise and reproducible induction of RPE cell oxidative damage and apoptosis, followed by secondary photoreceptor degeneration—processes that recapitulate the core pathological cascade of human AMD (Énzsöly et al., 2014; Yang et al., 2021). This method, characterized by acute and single-factor induction, fails to recapitulate the chronic, age-related, multifactorial-driven complexity of human AMD. In the future, it is necessary to further validate in combination with gene-editing models or laser-induced models. It does not recapitulate early features (e.g., drusen) or induce neovascularization, so findings should be cautiously extrapolated to human AMD. Third, although the putative targets identified via network analysis were validated at the mRNA level *in vitro*, this validation is limited. The network analysis itself is hypothesis-generating, and false positives cannot be ruled out; thus, we plan to conduct protein-level validation (e.g., Western blotting) and functional studies (e.g., target knockout) for each candidate in future research.

## 5 Conclusion

This study demonstrates through *in vitro* and *in vivo* models that DHM significantly ameliorates SI-induced retinal damage. Its protective effects encompass suppression of RPE apoptosis and preservation of retinal architecture. Network analysis and molecular docking predicted seven putative targets, with enrichment analyses revealing these targets’ modulation of key AMD-associated pathological pathways—including angiogenesis (e.g., *VEGFA* pathway), apoptosis (e.g., *BCL2* pathway), and extracellular matrix metabolism (e.g., *MMP2/MMP9* pathway). It must be emphasized that these computational predictions require experimental validation. Our findings provide preliminary evidence supporting DHM as a potential multi-target prophylactic/therapeutic candidate for AMD. Nevertheless, its precise mechanisms of action and clinical translatability warrant further in-depth investigation.

## Data Availability

The original contributions presented in the study are included in the article/Supplementary Material, further inquiries can be directed to the corresponding author.
