# Supplementary material for: Dihydromyricetin attenuates age-related macular degeneration: pharmacological effects and exploration of putative targets
Source: Front Pharmacol. 2025 Aug 21;16:1588970. doi: 10.3389/fphar.2025.1588970 (PMC12408514; doi:10.3389/fphar.2025.1588970)
Supplement: Supplementary file 2 [file Table1.docx]

| ID | 5’-3’ | 3’-5’ |
| --- | --- | --- |
| VEGFA | TCTTCAAGCCATCCTGTGTG | ATCCGCATAATCTGCATGGT |
| MAPT | GACGCTGGCCTGAAAGAATC | CCGCTGTTGGAGTGCTCTTA |
| APP | CCGCTGCTTAGTTGGTGAGTTTGT | ACGGTGTGCCAGTGAAGATGAGTT |
| KDR | TGACAACCAGACGGACAGTG | GGGCACCATTCCACCAAAAG |
| MMP2 | GACCGCTTGGCTTCAAATCA | GGTGTTCAGGTATTGCATGTG |
| MMP9 | CGCAGACATCGTCATCCAGT | AACCGAGTTGGAACCACGAC |
| HIF1A | AGCTTGCTCATCAGTTGCCA | CCAGAAGTTTCCTCACACGC |
| PGF | AATGTCACCATGCAGCTCCT | TCTCTCTCCTCCAAGGGGTG |
| BCL2 | GTCATGTGTGTGGAGAGCGT | AGTTCCACAAAGGCATCCCAG |
| PPARG | CTGCTTAATTCCCTTTCC | AGGAGTGGGAGTGGTCTTCC |
| ICAM- 1 | AGTGACCATCTACAGCTTTCCG | CCCCATTCAGCGTCACCTT |
| APOE | AACTGGCACTGGGTCGCTTT | GCCTTCAACTCCTTCATGGTCTCGT |
| APP | CCGCTGCTTAGTTGGTGAGTTTGT | ACGGTGTGCCAGTGAAGATGAGTT |
| ABCA4 | AGCTGAAGAAGGCTGTGAGCTT | TGGGACTGTTCCCGATGTTG |
| HTRA1 | TGTCACTCACGTCCAGCAAA | GACATCATTGGCGGAGACCA |
| TERT | AAATGCGGCCCCTGTTTCT | CAGTGCGTCTTGAGGAGCA |
| DNMT1 | CCGACTACATCAAAGGCAGC | AGGTTGATGTCTGCGTGGTA |
| GAPDH | TGACTTCAACAGCGACACCCA | CACCCTGTTGCTGTAGCCAAA |
