# Supplementary material for: Dihydromyricetin attenuates age-related macular degeneration: pharmacological effects and exploration of putative targets
Source: Front Pharmacol. 2025 Aug 21;16:1588970. doi: 10.3389/fphar.2025.1588970 (PMC12408514; doi:10.3389/fphar.2025.1588970)
Supplement: Supplementary file 3 [file Table2.docx]

| ID | Cat | Producer |
| --- | --- | --- |
| Phospho-p53 | 2521 | Cell Signaling Technology |
| Bax | 2772 | Cell Signaling Technology |
| Bcl-2 | 3498 | Cell Signaling Technology |
| Cleaved Caspase-3 | 9661 | Cell Signaling Technology |
| β-Actin | 4967 | Cell Signaling Technology |
| IgG(Rabbit) | 7074 | Cell Signaling Technology |
